# Leveraging ultrashort echo time magnetic resonance imaging to quantify sex-specific adaptations to the glenoid labrum across the lifespan

**DOI:** 10.1038/s41598-026-60700-w

**Published:** 2026-07-28

**Authors:** Colleen M. Vogel, Kwan-Jin Jung, Denali M. Hutzelmann, Heath B. Henninger, Joshua M. Leonardis

**Affiliations:** 1 College of Applied Health Sciences, University of Illinois Urbana-Champaign, Urbana, IL USA; 2 Beckman Institute for Advanced Science and Technology, University of Illinois Urbana-Champaign, Urbana, IL USA; 3 Department of Orthopaedics, University of Utah, Salt Lake City, UT USA; 4 School of Health and Rehabilitation Sciences, College of Medicine, The Ohio State University, 453 West 10th Avenue, Columbus, OH 43210 USA

**Keywords:** Anatomy, Biomarkers, Diseases, Medical research

## Abstract

The glenoid labrum is a collagen‑rich fibrocartilage structure critical to shoulder stability yet susceptible to age‑related degeneration. Quantitative, *in‑vivo* assessment of its composition remains limited. Ultrashort echo time MRI with magnetization transfer (UTE‑MT) enables estimation of macromolecular fraction (MMF), a biomarker sensitive to the collagen‑rich matrix content of the glenoid labrum. This study investigated the effect of age, sex, menopause status, and arm dominance on the glenoid labrum MMF. Forty‑two healthy adults (25 females, mean age: 47 ± 15 years; 17 males, mean age: 38 ± 15 years) underwent bilateral shoulder MRI on a 3T system using a double echo steady state sequence (DESS) for segmentation and a custom UTE‑MT protocol to quantify MMF. Labra were segmented from DESS images, and voxel‑wise MMF was computed. Mean MMF was calculated for each arm. A linear mixed-effects model with a random intercept for subject to account for within-subject dependence tested the effects of age, sex, arm dominance, and their respective interactions. A linear mixed effect model with a random intercept for subject tested the effect of menopause status among females on mean MMF. Males exhibited higher mean labral MMF than females (male: 40.4 ± 7.3%, female: 39.6 ± 7.2%; *p* = 0.016). Age showed no main effect on mean MMF. However, a significant sex‑by‑age interaction indicated a steeper age‑related decline in males (*p* = 0.0113). There was no significant effect of arm dominance on mean MMF (dominant: 41.1 ± 7.6%, non-dominant: 38.8 ± 6.9%; *p* = 0.412), and there was no significance found for the interactions of dominance and sex, dominance and age, or dominance, sex, and age. Among females, mean MMF did not differ by menopause status (premenopausal 39.4 ± 7.4% vs. postmenopausal 39.7 ± 7.1%; *p* = 0.761). UTE‑MT MRI detects sex‑specific differences in glenoid labrum MMF, with a significant sex‑by‑age interaction despite no main effect of age. These findings support MMF’s potential as a non‑invasive quantitative marker of labral composition and motivates longitudinal studies to define clinically meaningful thresholds and regional variation.

## Introduction

The shoulder joint complex is designed to maintain stability across a large range of motion. The glenohumeral joint features a shallow glenoid fossa upon which the humeral head articulates, enabling extensive movement across all degrees of freedom. The mobility of the glenohumeral joint is supported by dynamic (glenohumeral and scapulothoracic musculature) and static stabilizers (glenohumeral ligaments, joint capsule, labrum), which collectively serve to maintain joint stability^1^. Among these soft tissues, the glenoid labrum plays a particularly vital role in maintaining both static and dynamic joint stability by increasing glenoid congruency and anchoring the joint capsule. Because the labrum is heavily relied upon during the regular performance of upper extremity activities of daily living, it is vulnerable to cumulative stress and degeneration, making it a common site of injury, including superior labrum anterior to posterior (SLAP) tears.

The glenoid labrum is a ring of densely packed fibrocartilage that increases glenoid fossa depth, aiding in joint stability by passively resisting humeral head migration^2,3^. It is composed of predominantly type I collagen fibers arranged in a heterogeneous pattern, with some regions integrating with the joint capsule and others blending into the origin of the long head of the biceps brachii tendon^4,5^. Anterior and superior regions of the labrum tend to be more fibrous (more type I collagen), while the posterior and inferior regions contain more fibrocartilaginous elements (combination of type I and II cartilage)^6^. This composition allows the labrum to effectively resist multidirectional forces imposed during arm movements and weight-bearing activities^7^. However, the regional variation in labral mechanical properties associated with regional variation in composition contributes to region-specific injuries including Bankart lesions (anteroinferior portion) and SLAP tears (superior portion). Damage to the labrum can occur from trauma, overuse, or age-related degeneration and leads to pain, instability, and significant upper limb functional impairment. Age-related labrum degeneration may involve fraying and diminished vascularity, thinning of the fibrocartilage, and collagen degradation^8,6,9^.

Age-related collagen degradation can occur when matrix metalloproteinases (MMPs) enter the extracellular matrix of collagenous tissues^10–12^. MMPs regulated by cytokines act as pro-inflammatory mediators, prompting MMP gene expression when the immune system detects inflammation. Therefore, an increase in cytokines can lead to the increase of MMP expression, prompting collagen degradation^13^. With excessive loading and chronic overuse, MMP expression increases while collagen synthesis decreases, resulting in greater collagen degradation than deposition (Chen et al., 1999; Forsyth et al., 2005). Similarly, the natural aging process contributes to collagen degradation through mechanisms comparable to those induced by overuse^14^. In the meniscus of the knee, older adults possess less type I collagen when compared to younger adults^15,16^. This decrease of collagen with age may contribute to meniscal degeneration, which is strongly associated with osteoarthritis (Zhang et al.,^17^. Fibrosis of fibrocartilage, the most collagen-rich form of cartilage, has been associated with aging in the upper extremity as well^18,19^. Increased MMP expression can lead to fibrotic tissue formation in certain pathologies including in adhesive capsulitis, which has a mean onset age of 55^20^. Therefore, a quantitative assessment of the microstructural changes to the labrum across the lifespan may provide valuable insight into normal and potentially pathological adaptations.

While imaging modalities play a crucial role in diagnosing shoulder pathology and monitoring known disease progression, they have traditionally fallen short in their ability to capture underlying, preclinical structural changes to musculoskeletal tissues. Magnetic resonance imaging (MRI) provides high-resolution visualization of soft tissue structures, allowing for the evaluation of labral composition^21^. Advanced quantitative MRI techniques such as T2 mapping have been utilized to detect early degenerative changes in knee cartilage, showing that T2 relaxation times increase with decreasing tissue density and increasing disorganization^22,23^. However, T2 mapping has limitations, including the magic angle effect in which signal alterations occur depending on the collagen fiber orientation relative to the magnetic field, potentially confounding the interpretation of results gathered using this approach^24^. Ultrashort echo time (UTE) sequences enable the evaluation of tissues with very short relaxation times, including collagen-rich matrix structures like the glenoid labrum^25,26^. UTE with magnetization transfer (MT) is useful for enhancing image contrast by exploiting the exchange of magnetization that naturally occurs between water and macromolecules in tissue^27^. Macromolecular fraction (MMF) can be obtained using this approach, which demonstrates the fraction of macromolecules like collagen-rich matrices that are present within a tissue^23^. In the context of collagen-rich tissues, increasing MMF indirectly suggests higher collagen content within the extracellular matrix, which at extreme levels may be consistent with fibrosis while decreasing MMF may indicate collagen degradation. Compared to T2 relaxation, which predominantly reflects water content and is influenced by tissue hydration and collagen fiber orientation, MMF allows for the non-invasive in vivo quantitation of macromolecular density and collagen-rich matrix^27^. Additionally, UTE-MT sequences minimize the magic angle effect, making them especially valuable for evaluating fibrocartilaginous tissues such as the glenoid labrum^28^. While UTE has not been adopted into clinical routine imaging, support for its use in musculoskeletal contexts is growing^29^; Zhang et al.,^17^. Given its previous application to analogous structures such as the meniscus, UTE-MT may offer valuable insight into individual differences and compositional changes over time in the glenoid labrum^30^.

Therefore, the primary purpose of this study was to leverage UTE-MT to quantify the effect of age on glenoid labrum composition while accounting for the known covariates of biological sex and arm dominance. We tested the primary hypothesis that (1) MMF differs between sexes and decreases with age, reflecting known sex-based differences in connective tissue composition and the progressive tissue degradation associated with aging. We tested the secondary hypothesis that (2) arm dominance would impact MMF. Finally, we explored the effect of menopause on labrum MMF, as menopause-related hormonal changes influence collagen-rich matrix metabolism and may be reflected in the glenoid labrum.

## Methods

### Participants

Forty-two individuals (25 F, 17 M, age: 45.3 ± 16.9 yrs) were included in this study (Table 1). Of the twenty-five females, thirteen were post-menopausal (having gone at least twelve consecutive months without menstruation). Participants were randomly sampled from the Champaign-Urbana, Illinois area. The first wave of recruitment focused on young adults and the second wave of recruitment expanded to the broader community of middle-aged and older adults. Data collection took place in the Biomedical Imaging Center at the Beckman Institute for Advanced Science and Technology on the University of Illinois campus. Individuals between the ages of 18 and 80 were eligible to participate in this study. Individuals who were pregnant, have/had upper extremity surgeries, any neurological conditions, nonremovable foreign metallic objects in the body (i.e. implanted mechanical or electrical devices), claustrophobia, uncontrollable shaking, or are unable to lie still for 1.5 h were excluded. Written informed consent was obtained prior to the collection of any data. All study procedures were approved by the University of Illinois Institutional Review Board (IRB23-0379) and were performed in accordance with the Declaration of Helsinki.

**Table 1:** Participant Demographics (Mean ± SD). Note that the pre- and postmenopausal groups are subdivisions of the female participants.

Age (yrs)	Males (*n* = 17)	Females (*n* = 25)	Premenopausal (*n* = 12)	Postmenopausal (*n* = 13)
	38 ± 15	47 ± 15	35 ± 12	59 ± 4
Height (cm)	179 ± 6	165 ± 6	168 ± 6	163 ± 6
Weight (kg)	82 ± 11	68 ± 9	69 ± 11	69 ± 7
BMI (kg/m^2^)	26 ± 2	25 ± 4	25 ± 5	26 ± 2

### Procedures

All participants underwent bilateral magnetic resonance imaging (3T MAGNETOM Prisma-Fit, Siemens) of the upper extremities using a combination of small and large flex coils. The participants laid supine with their arms at their sides, and elbows extended. Additional cushioning under the knees and in between the participants’ arms and machine were provided for comfort and to minimize movement.

A double echo steady-state sequence (DESS) (TE: 5.40ms, TR: 16.00ms, flip angle: 25°, field of view: 176 mm, 0.6 mm isotropic voxel size, scan time: 5:00 per side) was performed and utilized for tissue segmentation and landmark identification. A custom 3D UTE-MT sequence (TE1: 0.08ms, TE2: 4.52ms, TR: 45.00ms, field of view: 216 mm, 1.0 mm isotropic voxel size) was acquired to calculate the magnetization transfer and estimate MMF through UTE-MT modelling. This sequence was designed to achieve UTE using nonselective radiofrequency pulse followed immediately by readout gradients. The readout trajectory combined radial and spiral sampling^31^. This 2D hybrid trajectory was divided into 48 interleaves to cover the in-plane k-space and the resulting trajectory disc was rotated about the Z-axis in 238 angular steps to sample the full 3D k-space^32^. Magnetization saturation was performed using two Gaussian RF pulses (duration: 3840 µs, flip angle: 270°, frequency offset: 1500 Hz), each followed by a spoiling gradient. After saturation, a low-flip angle excitation (rectangular RF, 5°) and read out were repeated four times to shorten the overall scan time. Image reconstruction was carried out using the PICS algorithm implemented in the BART toolbox^33^.

### Data analysis

**Fig. 1:**
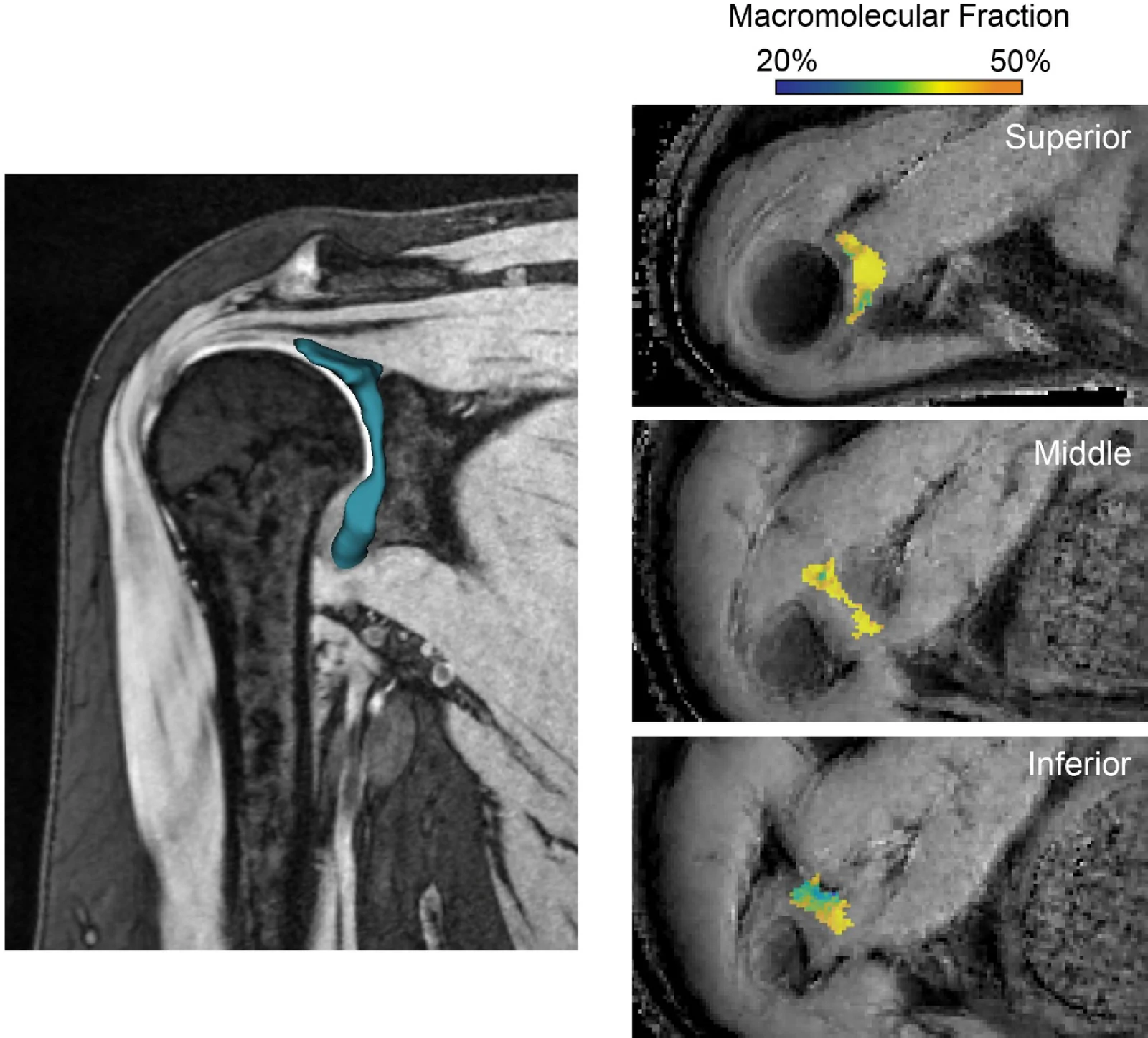
Representative DESS (left) and UTE‑MT (right) images from a single participant. Labral segmentation was performed volumetrically across the labrum and the MMF values are overlaid in transverse view slices from the superior, middle, and inferior regions. Glenoid labrum (MMF), represented by color intensity corresponding to collagen-rich matrix values, is overlaid on each slice to illustrate variations in tissue composition.

One individual blinded to participant characteristics was responsible segmenting tissues, landmarking, and registering images. UTE-MT scans were registered to DESS images with a semi-automated landmark registration (3D Slicer,^34^ using eight anatomical landmarks on the scapula and humerus (anterior, posterior, superior, and inferior glenoid rim, infraglenoid tubercle, neck of the scapula, greater tubercle of the humerus, and most superior point of the humeral head). From DESS axial, sagittal, and coronal images, one investigator segmented the labrum using built in semi-automated functions^34^. Volumes were eroded by 1 voxel at the margins in-plane using Simple ITK to avoid inclusion of nearby structures and mitigate partial volume effects. Custom analyses in MATLAB (version R2024b, Mathworks, Natick, MA, USA) quantified voxel-by-voxel labrum MMF using three-dimensional models rendered from DESS images (Fig. 1). Mean MMF was calculated bilaterally for each participant.

### Statistical analysis

All statistical analyses were performed in R (R Core Team, version 4.4.3). Because each participant contributed bilateral shoulder metrics, all analyses utilized linear mixed-effects intercept models with a random intercept for subjects to account for within-subject dependence. To explicitly model the paired, within-subjects nature of the bilateral data and simultaneously evaluate our primary and secondary hypothesis, mean labrum MMF was modeled as the outcome with age (continuous), sex (female, male), arm dominance (dominant, non-dominant), and all respective interactions specified as fixed factors. This simultaneous approach allowed us to evaluate the independent effects of age, sex, and laterality/arm dominance while rigorously controlling for Type I error inflation and potential confounding across predictors. Our exploratory hypothesis investigated the effect of menopause on mean MMF in the labrum using a separate linear mixed model with a random intercept for subject, mean MMF as the outcome and age and menopause (pre-/post-menopause) status as factors. Partial eta squared (η_p_²) was used to estimate effect size, representing the proportion of variance in the dependent variable attributable to a given factor after accounting for other variables in the model. In general, ηp² values of approximately 0.01, 0.06, and 0.14 are interpreted as small, medium, and large effects, respectively.

## Results

**Fig. 2:**
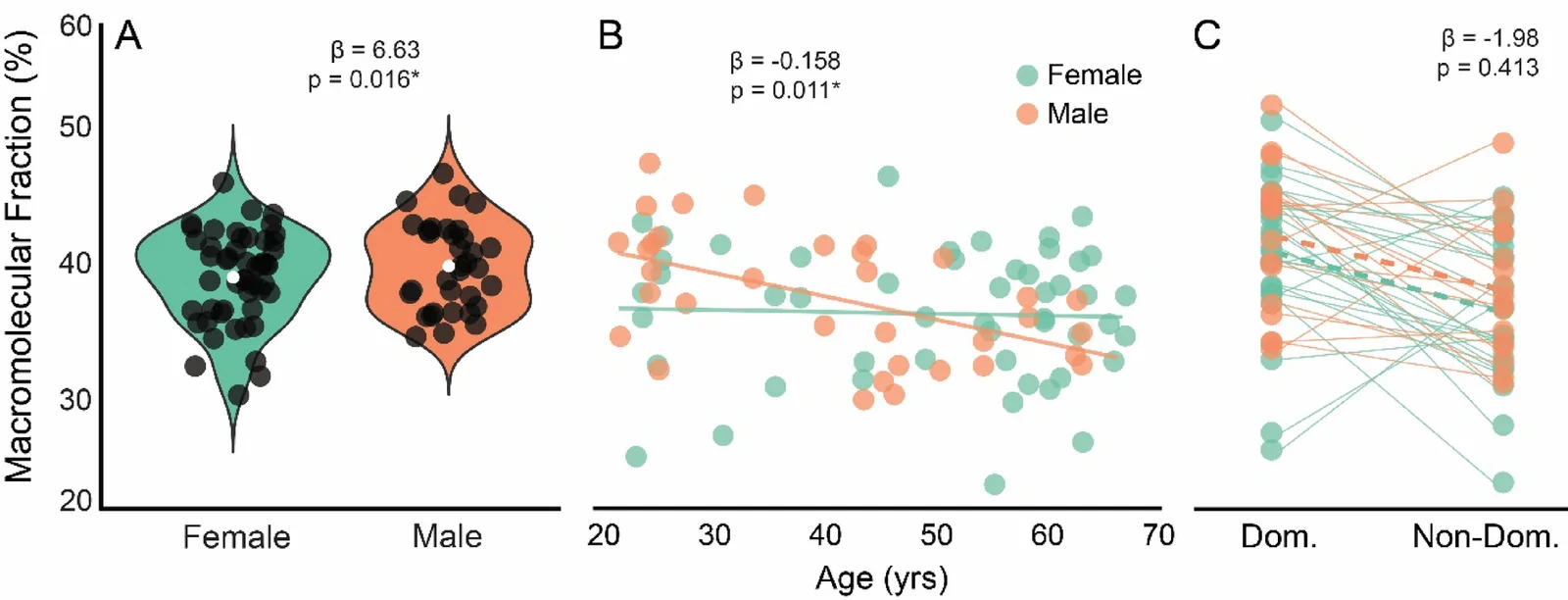
(**A**) Violin plots representing mean (white dot) and probability densities for male and female glenoid labrum macromolecular fraction (MMF) without controlling for arm dominance. Individual participant data are presented by opaque dots. Asterisk indicates significant effect of sex. (**B**) Scatterplot illustrating the interaction between sex and age on average glenoid labrum MMF. Asterisk indicates significant interaction effect. (**C**) Paired scatterplot illustrating within-subject glenoid labrum MMF variation by arm dominance and sex.

**Fig. 3:**
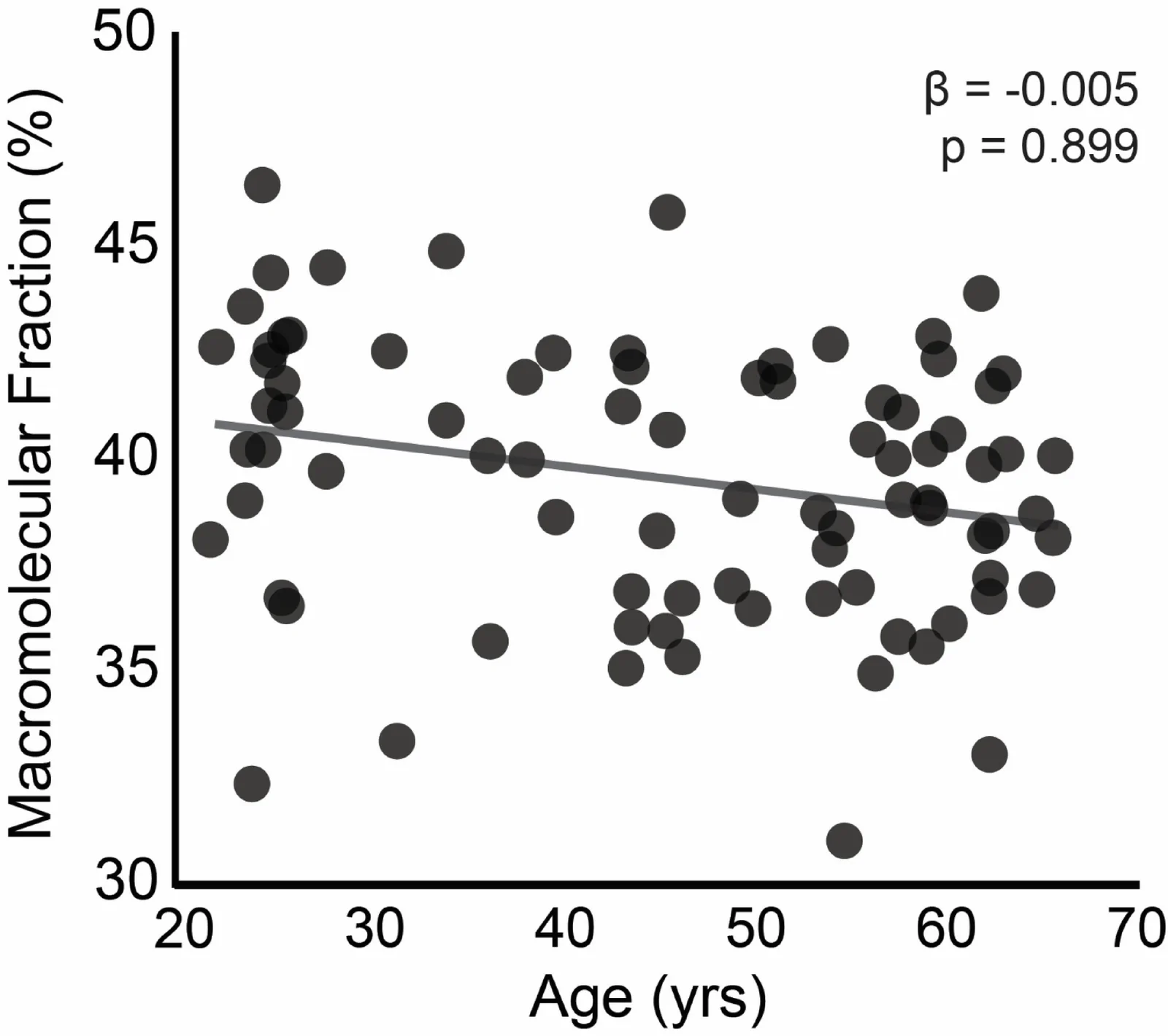
Scatterplot illustrating the relationship between glenoid labrum macromolecular fraction (MMF) and age, independent of sex or arm dominance. Opaque dots represent individual arms within participants. Best-fit least squares line illustrates the relationship between our variables.

Mean labral MMF values are presented in Table 2. There was a significant main effect of sex on mean MMF with males exhibiting greater mean MMF (40.4 ± 7.3%) than females (39.6 ± 7.2%) (F_1,76_=6.070, β = 6.630, 95% CI [1.270, 11.99], *p* = 0.016, η_p_^2^ = 0.074) (Fig. 2A). Age did not have a significant effect on mean MMF (F_1, 76_=0.016, β=-0.005, 95% CI [-0.081, 0.071], *p* = 0.8998, η_p_^2^ = 0.0002) (Fig. 3). However, the interaction of sex and age showed significance (F_1,76_=6.7347, β=-0.158, 95% CI [-0.280, -0.037], *p* = 0.0113, η_p_^2^ = 0.0814), where mean MMF declined more steeply with age among males than females (Table 2; Fig. 2B).

**Table 2:** Macromolecular fraction (Mean ± SD) across sex, menopause status (pre- vs. post), and arm dominance (dominant (Dom) vs. non-dominant (Non)).

	Males	Females	Pre	Post	Dom	Non
MMF (%)	40.4 ± 7.3	39.6 ± 7.2	39.4 ± 7.4	39.7 ± 7.1	41.1 ± 7.6	38.8 ± 6.9

**Fig. 4:**
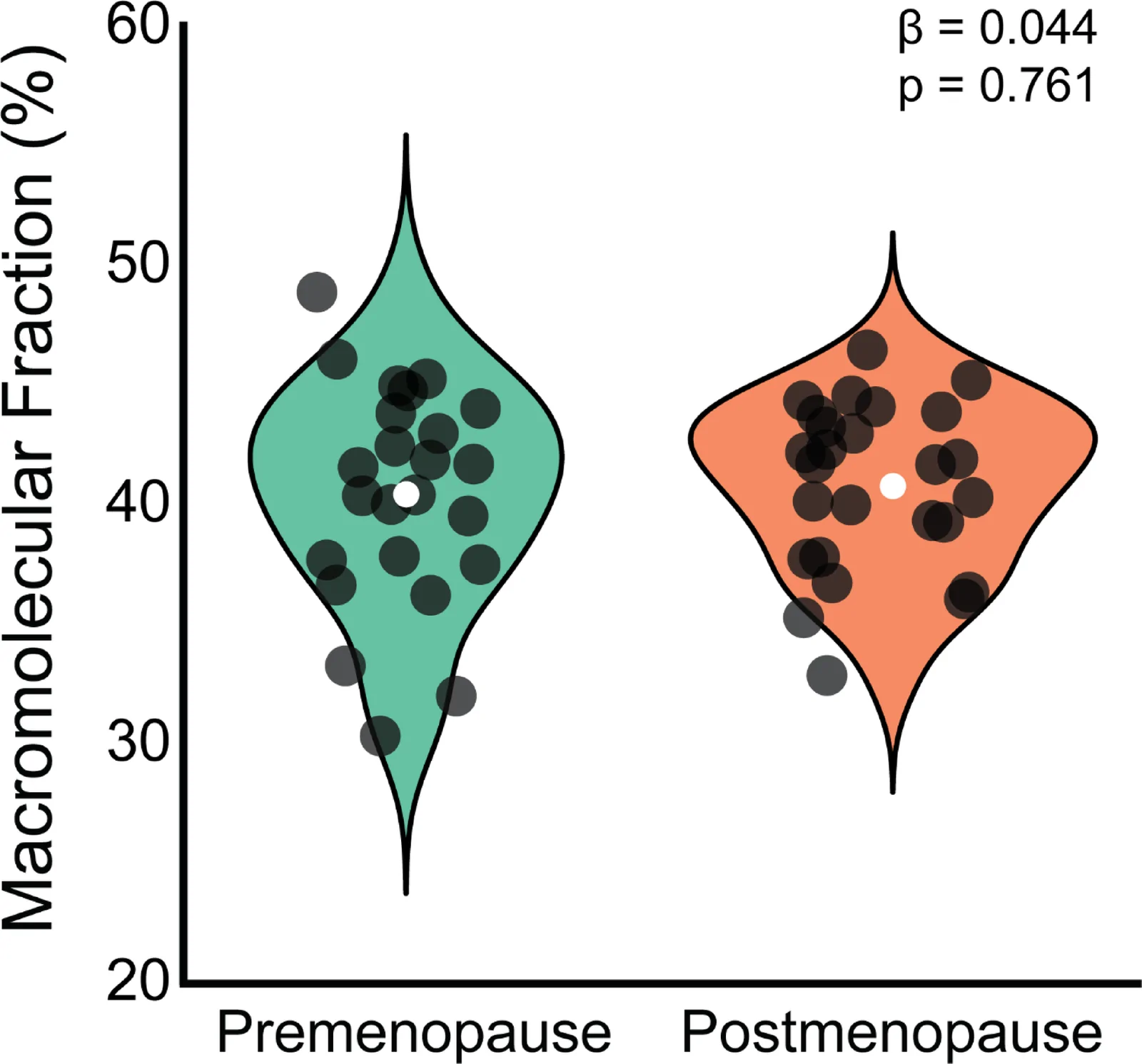
Violin plots representing mean (white dot) and probability densities for pre- and post- menopausal female glenoid labrum macromolecular fraction (MMF) without controlling for arm dominance. Individual participant data are presented by opaque dots.

There was no significant main effect of arm dominance on MMF (dominant: 41.1 ± 7.6%, non-dominant: 38.8 ± 6.9%; F_1,76_=0.6783, β=− 1.981, 95% CI [− 6.773, 2.810], *p* = 0.4127, η_p_^2^ = 0.0088). The interactions between arm dominance and sex (F_1,76_=1.2845, β=− 3.894, 95% CI [− 10.74, 2.950], *p* = 0.2606, η_p_^2^ = 0.0166), dominance and age (F_1,76_=0.033, β=− 0.009, 95% CI [− 0.106, 0.088], *p* = 0.8554, η_p_^2^ = 0.0004)) (Fig. 2C), and dominance, sex, and age (F_1,76_=1.9128, β = 0.108, 95% CI [− 0.47, 2.63], *p* = 0.1707, η_p_^2^ = 0.0246) were not significant. We failed to detect a main effect of menopause status on mean MMF (F_1, 48_=0.094, β = 0.04, 95% CI [− 1.624,2.232], *p* = 0.76, η_p_^2^ = 0.019), indicating no meaningful difference between premenopausal (39.4 ± 7.4%) and postmenopausal (39.7 ± 7.1%) females (Fig. 4).

## Discussion

The purpose of this study was to determine the impact of aging on the composition of the glenoid labrum using UTE-MT. We hypothesized that (1) MMF decreases with age and differs between sexes and (2) MMF differs between the dominant and non-dominant arms. We also explored the possible impact that menopause status may have on labral composition. Our findings suggest that sex significantly influenced mean MMF, with males demonstrating higher values than females, and a notable sex-by-age interaction indicated a steeper decrease in MMF with age among males. We did not observe arm dominance as a significant factor, and arm dominance did not differ by sex. We found that menopause status had no significant effect on mean MMF. To our knowledge, this study is the first to utilize UTE-MT to explore the impact of age, sex, and arm dominance on the glenoid labrum. Our findings add to the growing body of UTE-MT research and the utilization of MMF to evaluate musculoskeletal tissues.

UTE-MT has been utilized to examine the composition of the Achilles (19–22% MMF)^35,36^, tibialis (anterior tibialis tendon: 17% MMF; posterior tibialis tendon: 21% MMF)^37^, rotator cuff tendons (supraspinatus and infraspinatus: 11.61–15.86% MMF)^38^ and the tibial meniscus (15.84–18.19% MMF) (Zhang et al.,^17^. Despite the similar composition of the meniscus and glenoid labrum, our study found the glenoid labrum to have much greater MMF values (30.9–47.1% MMF). Tissue histology and the unique role of the tibial meniscus and glenoid labrum indicate why this discrepancy might exist. The structural purposes of the meniscus and labrum are to undergo loading and aid in joint stability. However, the primary role of the meniscus is shock absorption, as it undergoes near constant loading and its greater water content aids in viscoelastic load transmission^39^. The labrum has the primary role of stability, as it increases the congruency in the glenoid socket and helps with resisting excessive humeral head translation with a denser extracellular matrix^40^. Therefore, it is reasonable to believe the glenoid labrum, heavily comprised of collagenous fibrocartilage, has a greater collagen-rich matrix, reflecting higher MMF values. While the extracellular matrix of the meniscus is composed of 72% water and 22% collagen (type I and II)^41^, the composition of the extracellular matrix in the glenoid labrum is not well defined. Tissue hydration, heterogeneity, and partial volume may be potential confounder influencing MMF values. The glenoid labrum is characterized by its fibrocartilaginous composition (primarily type I collagen)^3^. Prior histological work has reported a dense ECM in the superior portion of the glenoid labrum (56.8±6.9%)^42^, suggesting that regional differences in matrix compositions may exist. Given this denser ECM, it is feasible that the superior portion of the labrum may have lower total water content. However, a subregional analysis was not performed due to the inability to reliably segment the inferior labrum for each individual. Therefore, the whole labrum was analyzed to maintain consistency across all participants and minimize segmentation-related bias. Future studies with increased imaging resolution may allow for assessment of regional MMF differences.

Sex-specific age-related collagen degeneration in musculoskeletal structures, which would be reflected by lower MMF, has significant implications for health and function. Although age-related changes in MMF have been observed in other musculoskeletal tissues such as the anterior and posterior tibialis tendons^37^, our study did not demonstrate age as a significant main effect on labral MMF. However, we did observe a significant sex-by-age interaction indicating that MMF values in males declined steeper with age. There is limited research considering sex-dependent lifelong aging effects on fibrocartilage and more research in this area is needed to conclusively identify the role of aging in fibrocartilage composition. Age-related degeneration has functional impacts on the mechanical properties of tissues like the meniscus or labrum. Loading the meniscus in adults over 75 does not elicit the same deformation-recovery observed in adults 65 and under, likely due to the age-related loss of collagen^43^. The labrum experiences similarly significant loading, and early age onset histopathological changes to the labrum tend to occur in regions where it experiences greater loading^7^. These changes are apparent in individuals above 30 years of age when it is common for the labrum to start showing signs of fraying^44^. While our findings failed to identify generalized age-related decline in labral MMF values, they do suggest that age-related compositional changes occur in a sex-specific manner.

While our work did not account for or identify glenoid labrum pathology, our results may suggest that naturally diminishing collagen-rich matrix occurs over the lifespan in some individuals which could contribute to the development of pathologies in later life. Because the labrum serves as an insertion site for the joint capsule and glenohumeral ligaments, age-related labral degeneration, such as decreased vascularity with aging^44^ and greater prevalence of SLAP tears over the age of 50^45^, may also affect surrounding structures. Age-related muscle atrophy may also factor into labral loading as the rotator cuff’s role of actively resisting glenohumeral translation can decline with age, causing increased reliance on the passive labrum to withstand loading^46,47^. Understanding the dynamic synergism between the glenoid labrum and nearby structures may help our understanding of the mechanisms in which labral, capsular, and ligamentous injuries or degeneration occur.

There are biological differences between the sexes that may also influence glenoid labrum structure and function. In our study, males exhibited a higher mean MMF than females. While it remains unclear what underlying mechanisms drive this difference, it is reasonable to consider hormones and hormonal variations playing a role between the sexes. The estrogenic influence on tissue health has been demonstrated in tendons, ligaments, and cartilage. Young females exhibit lower tendon stiffness and greater joint laxity than young males^48,49^. Estrogen can influence tendon compliance, as Achilles tendon strain decreases in long term oral-contraceptive users compared to non-users^50^. Greater ligamentous laxity predisposes females to greater anterior cruciate ligament injuries^51^. Cartilage is also sensitive to estrogen due to the expression of estrogen receptors that manage matrix turnover within the tissue^52^. Research on sex differences in the severity of rheumatoid arthritis suggests that estrogen may help protect against chondrocyte degeneration, hence lower disease severity in females^17^. Collectively, these estrogenic effects on tissues may contribute to sex-related differences in labral composition. However, these effects are unlikely to be explained solely by sex as estrogen concentration, receptor activity, physical activity level, and mechanical loading can change across the lifespan. We observed mean male MMF decrease more steeply with age when compared to females, demonstrating that sex may be a factor in the labral aging process. Therefore, changes in sex-specific hormones and loading mechanics may be important factors to consider when evaluating compositional changes in the labrum over the lifespan.

In the articular cartilage of the knee, an increased expression of estrogen receptors was shown in postmenopausal and osteoarthritic individuals, indicating greater cartilage deterioration and decreased collagen^53^. Previous work suggests that chondrocytes respond differently to estradiol, a type of estrogen, between sexes, with females showing greater sensitivity^54^. The change in estrogen form with age may also play a contributing role. As females age, estrogen is still being produced but the concentration and its effects may change. While estrogen aids in protection against deterioration, its defense is likely not as strong as it was premenopause^55^. For these reasons, we explored whether there would be a difference between MMF values in premenopausal and postmenopausal females as estrogen levels may factor in. We did not observe significant differences between the two (premenopausal: 39.4 ± 7.4%, postmenopausal: 39.7 ± 7.1%).

Although our findings did not demonstrate a significant effect of arm dominance on mean labral MMF (dominant: 41.1 ± 7.6%, non-dominant: 38.8 ± 6.9%), dominance-related loading may still be an important factor influencing labral health and composition. Prior studies have demonstrated that arm dominance and repetitive shoulder use is associated with structural differences in bone and soft tissue, particularly in athletic populations and occupations where upper extremity demands are high^56,57^. Our participants were sampled from the general population and do not necessarily reflect these extreme loading conditions. However, evidence specifically evaluating arm dominance and repetitive use and its influence on glenoid labrum structure and composition in the general population is limited. Despite this gap, mechanical loading is shown to be an important regulator of extracellular matrix responses in fibrocartilage^58^. While literature on glenoid labrum mechanobiology is limited, cells from the fibrocartilaginous acetabular labrum showed changes in extracellular matrix-related gene expression in response to cyclic stretching, including genes involved in collagen-rich tissue remodeling and matrix organization^59^. Similarly, tendon literature suggests an increase in collagen-rich matrix content with mechanical loading^60^. Given the similar fibrocartilaginous composition of the glenoid labrum, we expected load-mediated extracellular matrix responses to contribute to greater MMF values on the dominant side. Additionally, upper extremity dominance related differences may be more pronounced due to many upper extremity tasks being performed unilaterally^61^, whereas lower extremity dominance appears to be more task dependent^62^. While our findings did not support a significant dominance-related difference in the general population, it is possible that dominance may play a larger role in sub-populations where unilateral overhead use is more demanding.

The use of UTE-MT to assess healthy aging and potentially pathological degeneration of the labrum can be clinically advantageous. UTE scans are non-invasive, limiting patient complications while still gaining insight into tissue health. UTE paired with MT offers an objective biomarker of tissue composition. Its sensitivity in detecting early microstructural changes in macromolecular content before the presentation of gross morphological damage shows promising evidence for tracking degenerative changes over time, particularly in aging populations. With additional research to establish critical thresholds of change, clinicians may have an opportunity to intervene sooner, potentially delaying or preventing disease progression.

This study had several limitations, including the absence of a record of participants’ medical and pharmacological histories, and therefore lacked confirmed diagnoses and clinical radiologic assessments of our participants scans to rule out underlying and existing pathologies. To mitigate this limitation, we obtained an oral medical history as it relates to the upper extremity. Partial volume effects remain a limitation due to the thin structure of the labrum. Although segmentations were eroded by 1 pixel at the margins, residual partial volumes effects may still be present and influential in MMF values. No participants self-reported a diagnosis. However, some of the participants may have had undiagnosed labral pathology. Future work will account for pathological status and state. We were unable to obtain estrogen levels at the time of study although we did include classification of their reproductive stage of life. Our future work aims to include the analysis of serum biomarkers, specifically sex hormones, to better elucidate the role they play in the maintenance of labral health and composition. Similarly, we did not account for oral contraceptive use in females. We believe this is an important consideration for future research, as it likely impacts tissue health^63^. Although MMF can serve as a proxy for collagen quantification, the functional mechanical properties, such as strength and stiffness, cannot be assumed from the collagen-rich matrix alone. Formal segmentation reproducibility metrics were not calculated retrospectively. Finally, our convenience sample provided only a cross-sectional snapshot of labral health and composition in healthy individuals across much of the adult lifespan. While our findings cannot be generalized to longitudinal changes in labral health, it may support the use of MMF as a biomarker to track changes in tissue composition over time.

## Conclusion

In conclusion, this study presents a novel application of UTE-MT to evaluate the influence of age, sex, and arm dominance on the macromolecular matrix composition of the glenoid labrum. Males exhibited higher labral MMF values and a steeper age-related decrease in labral MMF. UTE-MT exhibits the potential to provide quantitative support to diagnostic imaging. Further research may investigate similar parameters in the surrounding tissues, such as the joint capsule and glenohumeral ligaments to establish a comprehensive evaluation of health and pathological age-related adaptations.

## Data Availability

The data that support the findings of this study are available upon reasonable request to J.L. Raw imaging data are not available due to the contained information that could compromise participant privacy.
